# Mining of high utility-probability sequential patterns from uncertain databases

**DOI:** 10.1371/journal.pone.0180931

**Published:** 2017-07-25

**Authors:** Binbin Zhang, Jerry Chun-Wei Lin, Philippe Fournier-Viger, Ting Li

**Affiliations:** 1 Department of Biochemistry and Molecular Biology, Health Science Center of Shenzhen University, Shenzhen, China; 2 School of Computer Science and Technology, Harbin Institute of Technology Shenzhen Graduate School, Shenzhen, China; 3 School of Natural Sciences and Humanities, Harbin Institute of Technology Shenzhen Graduate School, Shenzhen, China; Southwest University, CHINA

## Abstract

High-utility sequential pattern mining (HUSPM) has become an important issue in the field of data mining. Several HUSPM algorithms have been designed to mine high-utility sequential patterns (HUPSPs). They have been applied in several real-life situations such as for consumer behavior analysis and event detection in sensor networks. Nonetheless, most studies on HUSPM have focused on mining HUPSPs in precise data. But in real-life, uncertainty is an important factor as data is collected using various types of sensors that are more or less accurate. Hence, data collected in a real-life database can be annotated with existing probabilities. This paper presents a novel pattern mining framework called high utility-probability sequential pattern mining (HUPSPM) for mining high utility-probability sequential patterns (HUPSPs) in uncertain sequence databases. A baseline algorithm with three optional pruning strategies is presented to mine HUPSPs. Moroever, to speed up the mining process, a projection mechanism is designed to create a database projection for each processed sequence, which is smaller than the original database. Thus, the number of unpromising candidates can be greatly reduced, as well as the execution time for mining HUPSPs. Substantial experiments both on real-life and synthetic datasets show that the designed algorithm performs well in terms of runtime, number of candidates, memory usage, and scalability for different minimum utility and minimum probability thresholds.

## Introduction

Knowledge discovery in databases (KDD) [1–4] aims at finding useful or hidden information in data. Association Rule Mining (ARM) and Frequent Itemset Mining (FIM) are two sets of techniques that play an important role in KDD. They have been well-studied and have many applications. The Apriori algorithm [3] is the first algorithm for mining association rules (ARs). It uses a level-wise approach to explore the search space of patterns. In its first phase, Apriori relies on a minimum support threshold to discover frequent itemsets (FIs). In its second phase, Apriori combines the discovered FIs to obtain ARs respecting a given minimum confidence threshold. ARM and FIM only considers the occurrence frequency of items in a binary database. Thus, factors such as the profit, weight, and interestingness of patterns are not considered by the pattern discovery process of traditional ARM and FIM.

To address this limitation, the problem of High-Utility Itemset Mining (HUIM) [5–9] has been introduced. It is an extension of FIM that considers additional factors such as the unit profits of items and their quantities in transactions, to mine high-utility itemsets (HUIs). Many algorithms such as Two-Phase [5], HUP-tree [6], HUI-Miner [7] and FHM [8] have been studied to mine HUIs. Yun et al. [10] presented an incremental algorithm for handling the inserted transactions in the dynamic database for HUIM. Ryang and Yun [11] also proposed an algorithm for mining HUIs based on the data streams. An improved algorithm [12] of HUIM was also presented to speed up mining performance of HUIs. However, a drawback of these algorithms is that they do not consider the sequential ordering of items in transactions [4]. For instance, consider the sequential pattern <(*bread*), (*milk*), (*apple*)>, which indicates that some customer(s) bought the products *bread*, *milk* and *apple* in that order. HUIM techniques would simply ignore information about the ordering of items. To find patterns having a sequential ordering and reveal relationships between purchased items for customer behavior analysis, Sequential Pattern Mining (SPM) has been proposed. It aims at discovering the complete set of frequent sub-sequences that respect a minimum support threshold in a set of sequences of customer transactions. Agrawal et al. [13] proposed the AprioriAll, AprioriSome and DynamicSome algorithms to mine the set of sequential patterns (SPs). Other algorithms such as GSP [14], PrefixSpan [15] and SPAM [16] were also designed. Different from Apriori-based algorithms, which mines SPs using a level-wise approach, the SPADE [17] algorithm only needs three database scans to discover all SPs, while the FreeSpan [18] algorithm uses a pattern growth approach to mine the SPs.

Motivated by the needs of practical applications, high-utility sequential pattern mining (HUSPM) was introduced [19]. HUSPM is an extension of SPM, which consists of discovering sequential patterns having a high utility (e.g. yielding a high profit) in sequences. Such patterns have several applications such as for the analysis of customer shopping habits in retail stores. Contrarily to traditional SPM, HUSPM considers that each item is associated with a weight to indicate its relative importance (e.g weight, unit profit, interestingness), and each item has non-binary purchase quantities in sequences. A sequence is considered to be a high-utility sequential pattern (HUSP) if its utility is no less than a predefined minimum utility threshold (count), selected by the user. The task of HUSPM is more complex than that of traditional SPM since the downward closure (DC) property does not hold for the utility measure. For this reason, developing HUSPM algorithms requires to develop new strategies for reducing the search space that have not been previously used in SPM. Besides, HUSPM requires to consider the purchase order of items for mining HUSPs, which is non-trivial. Few studies [20–23] have been presented to mine HUSPs despite that mining HUSPs is desirable for several applications. To our knowledge, all these studies consider the discovery of HUSPs in precise data. But in real-life situations, data is often uncertain. For instance, sensors of a wireless network may collect data about the temperature and humidity in various locations. But, because these sensors are affected by various internal and external factors, readings can be inherently noisy and thus uncertainty values may be associated to each reading. To address the challenge of mining HUSPs in uncertain data, this paper proposes the task of High Utility-Probability Sequential Pattern Mining (HUPSPM) for mining High Utility-Probability Sequential Patterns (HUPSPs) in uncertain databases. The key contributions of this paper are the following.

1. A new framework called High Utility-Probability Sequential Pattern Mining (HUPSPM) is designed to handle the problem of high sequential pattern mining in uncertain databases. A baseline algorithm called HUSPM is introduced to mine the High Utility-Probability Sequential Patterns (HUPSPs) in uncertain databases. Based on the designed High Sequential-Weighted Utility-Probability Patterns (HSWUPs), a downward-closure property is obtained, and the correctness and completeness of the proposed algorithm for discovering HUPSPs is proven.
2. Three pruning strategies are respectively developed to reduce the search space by pruning unpromising candidate HUPSPs early. Results show that the proposed pruning strategies can speed up the mining performance, and that the number of candidates and memory usage can be greatly reduced.
3. An improved projection-based algorithm called P-HUSPM is designed to provide an efficient way of reducing the number of candidates for finding the HUPSPs. An experimental study show that this latter algorithm outperforms the baseline algorithm.

## Related work

Sequential pattern mining (SPM) [13] was introduced by Agrawal et al. They proposed the AprioriAll, AprioriSome and DynamicSome algorithms to discover the set of sequential patterns (SPs) in sequential databases. Srikant et al. then designed the GSP [14] algorithm, and Zaki et al. designed the SPADE [17] algorithm to mine SPs more efficiently. But these algorithms use a generate-and-test approach to mine SPs and several of them use a level-wise approach. As a result, these algorithms can produce a huge amount of candidate patterns. To address this problem, Pei et al. proposed the PrefixSpan [15] algorithm, which uses a pattern-growth approach to mine SPs. The SPAM [16] algorithm was then proposed, which performs a depth-first search to mine SPs. Although SPM has been widely used and applied in many applications, SPM algorithms can only handle itemsets or sequences from binary databases.

Frequent itemset mining in uncertain data (UFIM) has been well-studied. It has become an important research topic in recent years. Two main models have been developed, which are the expected support [24] and probabilistic frequentness [25] models. In the former model, an item/set is defined as a frequent itemset (FI) in an uncertain database if its expected support is no less than a predefined minimum support threshold. In the probabilistic frequentness model, an item/set is defined as an UFI if its frequentness probability is no less than a specified minimum probability. UApriori [24] is the first UFIM algorithm based on the expected support measure. Since the UApriori algorithm utilizes a level-wise approach, it still suffers from the problem of producing a huge amount of candidates. To avoid this problem, the UFP-growth [26], UH-mine [27] and CUFP-growth [28] algorithms were respectively proposed to mine FIs in uncertain databases using a pattern-growth approach. The UFP-growth and CUFP-growth algorithms utilizes a compact tree structure to discover UFIs, while the UH-mine algorithm adopts an hyper-structure for mining UFIs. The above algorithms are all based on the expected support model. Lee et al. [29] adopted the uncertain model to mine frequent itemsets with different item importance. Lee and Yun [30] then presented a minimum data structure without false positives for mining FIs.

As an alternative to the expected support model, Berbecker et al. [25] proposed the probabilistic frequentness model to mine UFIs. Sun et al. [31] proposed the p-Apriori and TODIS algorithms to respectively mine UFIs using bottom-up and top-down approaches. Tong et al. [32] compared algorithms using the expected support and probabilistic frequentness models. To mine Uncertain Frequent Sequential Patterns (UFSPs) in sequences, Muzammal et al. [33] proposed three algorithms relying on the expected support measure. Two algorithms were presented using the generate-and-test approach and one pattern-growth method was designed to mine UFSPs. Zhao et al. [34] established two uncertain sequence data models and proposed the U-PrefixSpan algorithm, which extends the famous PrefixSpan algorithm [15] to mine UFSPs using two different uncertain sequence data models.

In the past, the problem of High-Utility Sequential Pattern Mining (HUSPM) was introduced. The UWAS-tree and IUWAS-tree [19] algorithms were designed to use tree structures for handling the utility of web log sequences. The sequence-weighted utility (SWU) measure was proposed to maintain the downward closure (DC) property for mining HUSPs. The concept of SWU is similar to that of the TWU model [5] for high-utility itemset mining. The SWU of a pattern is defined as the sum of the utilities of all sequences containing the pattern. The SWU is used as an upper-bound on the utilities of patterns to obtain a downward closure property for mining HUSPs. Since the above two algorithms cannot deal with sequences containing multiple items in each sequence element (transaction), Ahmed et al. designed a level-wise UtilityLevel (UL) algorithm and a pattern-growth UtilitySpan (US) [20] algorithm to mine HUSPs. Yin et al. [21] then designed the LQS-tree structure to keep important information for mining HUSPs. Based on the LQS-tree structure, the USpan algorithm [21] adopts the SWU measure and the Sequence Weighted Downward Closure (SWDC) property to prune unpromising sequences and improve the performance of HUSP mining. Lan et al. [22] then proposed the projection-based high-utility sequential pattern mining (PHUS) algorithm for mining HUSPs with the maximum utility measure and a sequence-utility upper-bound (SUUB) model. The algorithm extends PrefixSpan [15] and uses a projection-based pruning strategy to obtain tight upper-bounds on sequence utilities to avoid considering too many candidates, and thus to improve the performance of mining HUSPs using the SUUB model. Alkan et al. [23] designed another upper-bound method called Cumulate Rest of Match (CRoM) and developed a Pruning Before Candidate Generation (PBCG) strategy to prune unpromising sequences for mining HUSPs.

## Preliminaries and problem statement

Let *I* = {*i*_1_, *i*_2_, …, *i*_*m*_} be a set of *m* distinct items. An uncertain quantitative sequence database is a set of sequences *USD* = {*S*_1_, *S*_2_, …, *S*_*n*_}, where each sequence *S*_*q*_ ∈ *USD* has a unique identifier *q*. A sequence *S*_*q*_ is defined as an ordered list of itemsets {*I*_1_, *I*_2_, …, *I*_*k*_}, where each itemset *I*_*r*_ is a set of items, where each item has a quantity *q*(*i*_*j*_, *I*_*r*_, *S*_*q*_) and a probability *p*(*i*_*j*_, *I*_*r*_, *S*_*q*_). Moreover, a profit table *ptable* = {*p*(*i*_1_), *p*(*i*_2_), …, *p*(*i*_*n*_)} indicates the unit profit values of each item. Two thresholds called the minimum expected support threshold and the minimum utility threshold, are respectively defined as *μ* and *ε*. For instance, consider the uncertain quantitative sequence database shown in Table 1, which will be used in the rest of this paper as running example. It contains 4 sequences and 6 items, represented by the letters (*a*) to (*f*). The corresponding unit profit table is shown in Table 2. In the running example, the minimum expected support threshold *μ* and the minimum utility threshold *ε* are set to *μ* (= 35%) and *ε* (= 27.7%), respectively.

**Table 1:** An uncertain quantitative sequence database.

SID	Sequence	Probability
*S*_1_	<(*a*, 3), (*b*, 4), [(*a*, 1), (*c*, 1), (*e*, 2)]>	0.6
*S*_2_	<[(*b*, 1), (*c*, 1)], [(*a*, 1), (*d*, 1)], [(*a*, 2), (*b*, 2)]>	0.8
*S*_3_	<(*f*, 1), [(*f*, 1), (*d*, 1)], [(*b*, 4), (*c*, 1)]>	0.5
*S*_4_	<(*b*, 1), (*c*, 1), [(*a*, 1), (*b*, 2)], [(*a*, 4), (*b*, 1)]>	0.9

**Table 2:** A unit profit table.

**Item**	*a*	*b*	*c*	*d*	*e*	*f*
**Profit**	2	1	3	4	1	2

**Definition 1** A sequence is called a *k*-sequence if the sequence contains *k* items.

For example, the sequence <(*a*), (*b*), (*a*, *c*, *e*)> is a 5-sequence since it contains 5 items, and the sequence <(*a*, *c*, *e*)> is a 3-sequence since it contains 3 items.

**Definition 2** The notation *S*_*a*_ ⊆ *S*_*b*_ indicates that a sequence *S*_*a*_ = <*I*_*a*1_, *I*_*a*2_, …, *I*_*an*_> is a sub-sequence of a sequence *S*_*b*_ = <*I*_*b*1_, *I*_*b*2_, …, *I*_*bm*_>.

For example, <(*a*), (*a*, *c*)> is a sub-sequence of the sequence <(*a*), (*b*), (*a*, *c*, *e*)> since (*a*)⊆ (*a*) and (*a*, *c*)⊆ (*a*, *c*, *e*).

The definitions of HUIM can be directly applied to SPM for high-utility sequential pattern mining (HUSPM) by considering both the quantities and unit profits of items in a database. The definitions are given next.

**Definition 3** The internal utility of an item (*i*_*j*_) in an itemset *I*_*k*_ of a sequence *S*_*q*_ is represented by *iu*(*i*_*j*_, *I*_*k*_, *S*_*q*_) (*i*_*j*_ ∈ *I*_*k*_, and *I*_*k*_ ∈ *S*_*q*_), and defined as:

$$\begin{array}{c} iu\left(i_{j},I_{k},S_{q}\right)=q\left(i_{j},I_{k},S_{q}\right)\times p\left(i_{j}\right). \end{array}$$ (1)

For example, the sequence *S*_2_ in Table 1 can be represented as <*I*_1_, *I*_2_, *I*_3_> and *I*_1_ = [(*b*, 1), (*c*, 1)], *I*_2_ = [(*a*, 1), (*d*, 1)], *I*_3_ = [(*a*, 2), (*b*, 2)]. The internal utility of (*a*) in *I*_1_ and *I*_2_ in *S*_2_ are respectively calculated as: *iu*(*a*, *I*_2_, *S*_2_) = 1 × 2 (= 2), and *iu*(*a*, *I*_3_, *S*_2_)= 2 × 2 (= 4).

**Definition 4** The maximum utility of an item in a sequence is the largest utility value for that item in that sequence. It is denoted as *imu*(*i*_*j*_, *S*_*q*_) and defined as:

$$\begin{array}{c} imu\left(i_{j},S_{q}\right)=max\left\{iu\left(i_{j},I_{k},S_{q}\right)|i_{j}\in I_{k}\wedge I_{k}\in S_{q}\right\}. \end{array}$$ (2)

For example in Table 1, (*a*) appears twice in *S*_2_. The maximum utility of (*a*) in S2 is calculated as: *max*{(*iu*(*a*, *I*_2_, *S*_2_) = 1 × 2 (= 2), *iu*(*a*, *I*_3_, *S*_2_) = 2 × 2 (= 4)}(= 4).

**Definition 5** The utility of a sub-sequence *S* in a sequence *S*_*q*_ is the maximum utility among the utilities of all occurrences of *S* in the sequence. It is denoted as *su*(*S*, *S*_*q*_).

For example in Table 1, the sub-sequence *S* = <(*b*), (*a*)> has two occurrences in *S*_2_. The utility values of the these two occurrences are respectively calculated as (1 × 1) + (1 × 2) (= 3) and (1 × 1) + (2 × 2) (= 5). Thus, the utility of the sub-sequence <(*b*), (*a*)> is calculated as *su*(<(*b*), (*a*)>, *S*_2_) = *max*(3, 5) (= 5).

In uncertain data mining, algorithms can be categorized as either using the expected support [24] or probabilistic frequentness [25] models. The expected support model used in the designed algorithm is defined as follows.

**Definition 6** The probability that a sequence *S* appears in a sequence *S*_*q*_ (*S* ⊆ *S*_*q*_) is denoted as *sp*(*S*, *S*_*q*_), where *sp*(*S*_*q*_, *S*) = *sp*(*S*_*q*_).

For example in Table 1, <(*a*)>⊆ *S*_1_ and <(*b*), (*d*)>⊆ *S*_1_. Moreover, *sp*(*S*_1_) (= 0.6), *sp*(*S*_2_) (= 0.8); *sp*(<(*a*)>, *S*_1_) (= 0.6) and *sp*(<(*b*), (*d*)>, *S*_2_) (= 0.8).

This paper combines the idea of high-utility sequential pattern mining with the expected support model to propose the novel problem of mining high utility-probability sequential patterns in uncertain databases. This problem is defined based on the following definitions.

**Definition 7** The utility and probability of a sequence *S* in an uncertain quantitative sequence database *USD* are respectively defined as:

$$\begin{array}{c} su\left(S\right)=\sum_{S\subseteq S_{q}\wedge S_{q}\in USD}su\left(S,S_{q}\right), \end{array}$$ (3)

$$\begin{array}{c} sp\left(S\right)=\sum_{S\subseteq S_{q}\wedge S_{q}\in USD}sp\left(S,S_{q}\right)=\sum_{S\subseteq S_{q}\wedge S_{q}\in USD}sp\left(S_{q}\right). \end{array}$$ (4)

For example in Table 1, a sequence *S* = <(*a*), (*b*)> is contained in *S*_1_, *S*_2_ and *S*_4_ where *su*(*S*, *S*_1_) (= 10), *su*(*S*, *S*_2_) (= 4), *su*(*S*, *S*_4_) (= 3) and *sp*(*S*, *S*_1_) = *sp*(*S*_1_) (= 0.6), *sp*(*S*, *S*_2_) = *sp*(*S*_2_) (= 0.8), *sp*(*S*, *S*_4_) = *sp*(*S*_4_) (= 0.9). Thus, the utility and probability of *S* are calculated as *su*(*S*) (= 10 + 4 + 3) (= 17) and *sp*(*S*) (= 0.6 + 0.8 + 0.9) (= 2.3), respectively.

**Definition 8** The utility of a sequence *S*_*q*_ in an uncertain quantitative sequence database *USD* is denoted as *SU*(*S*_*q*_) and defined as:

$$\begin{array}{c} SU\left(S_{q}\right)=\sum_{i_{j}\in I_{k}\wedge I_{k}\in S_{q}}su\left(i_{j},I_{k},S_{q}\right). \end{array}$$ (5)

For example in Table 1, the utility of *S*_1_ is calculated as *SU*(*S*_1_) (= 6 + 4 + 2 + 3 + 2) (= 17). To obtain a downward closure property, the sequence-weighted utility (SWU) is then utilized as an upper-bound on the utility of a sequence, which extends the TWU model [5] of HUIM.

**Definition 9** The sequence-weighted utility (*SWU*) of a sequence *S* in a database *USD* is the sum of the utilities of all sequences containing *S*, that is:

$$\begin{array}{c} SWU\left(S\right)=\sum_{S\subseteq S_{q}\wedge S_{q}\in USD}SU\left(S_{q}\right). \end{array}$$ (6)

For example in Table 1, the *SWU* of a sequence *S* = <(*a*), (*b*)> can be calculated as *SWU*(*S*) = *SU*(*S*_1_) + *SU*(*S*_2_) + *SU*(*S*_4_) (= 17 + 16 + 17) (= 50).

**Definition 10** The total utility of a sequence database *USD* is denoted as *TSU* and defined as the sum of the utilities of all sequences in *USD*:

$$\begin{array}{c} TSU=\sum_{S_{q}\in USD}SU\left(S_{q}\right). \end{array}$$ (7)

For example in Table 1, the sequence utilities of sequences _*S*1_ to *S*_4_ are respectively calculated as *SU*(*S*_1_) (= 17), *SU*(*S*_2_)(= 16), *SU*(*S*_3_)(= 15) and *SU*(*S*_4_)(= 17). Hence, the total utility of the uncertain sequential database is (17 + 16 + 15 + 17)(= 65).

**Definition 11** A sequence *S* in a database *USD* is defined as a High Utility-Probability Sequential Patterns (HUPSP) if 1) *sp*(*S*) ≥ |*USD*| × *μ*, and 2) *su*(*S*) ≥ *TSU* × *ε*.

In the designed algorithm, it recursively finds the set of HUPSPs with different lengths. For example, the set of 1-sequences is denoted as *HUPSPs*^1^ in the designed algorithm, and the set of 2-sequences is denoted as *HUPSPs*^2^ in the designed algorithm. Thus, the purpose of this paper is to recursively find the set of *k*-sequences (*k* ≥ 1, denoted as *HUPSPs*^*k*^) until no candidates are generated (the set of (*k*-1)-sequences (*HUPSPs*^*k*−1^) becomes ***null***).

**Problem Statement:** Based on the above definitions, the problem of mining high utility sequential patterns in uncertain databases is to discover the complete set of high utility-probability sequential patterns (HUPSPs). A sequence *S* is a HUPSP in an uncertain database if its probability is no less than the minimum expected support count and its utility is no less than the minimum utility count, that is:

$$\begin{array}{c} HUPSPs\leftarrow \{S|sp(S)\geq |USD|\times \mu \wedge su(S)\geq TSU\times \varepsilon \}. \end{array}$$ (8)

For instance, consider the database illustrated in Tables 1 and 2 for the running example. For a minimum expected support threshold *μ* of 35% and a minimum utility threshold *ε* of 27.7%, the set of HUPSPs is shown in Table 3.

**Table 3:** The derived HUPSPs for the running example.

Sequence	Expected Support	Utility	Sequence	Expected Support	Utility
<(*a*)>	2.3	18	<(*b*), (*a*), (*a*)>	1.7	18
<(*a*), (*a*)>	2.3	24	<(*c*), (*a*, *b*)>	1.7	21
<(*b*), (*a*)>	2.3	21	<(*c*), (*a*), (*a*)>	1.7	22
<(*c*), (*a*)>	1.7	18	<(*c*), (*a*), (*a*, *b*)>	1.7	25
<(*a*), (*a*, *b*)>	1.7	19	<(*b*), (*a*), (*a*, *b*)>	1.7	21
<(*b*), (*a*, *b*)>	1.7	18			

## Proposed algorithms and pruning strategies

### Proposed baseline algorithm

Several algorithms have been proposed to mine High-Utility Sequential Patterns (HUSPs) [19–21, 23] and High Probability Itemsets (HPIs) [35]. However, those two models cannot be directly combined for mining High Utility-Probability Sequential Patterns (HUPSPs). This paper addresses this issue by first presenting a baseline algorithm with three pruning strategies to discover the HUPSPs. Details are given below.

**Definition 12 (Matching sequence)** Let there be two sequence *S*_*a*_ = <*I*_1_, *I*_2_, …, *I*_*n*_> and *S*_*b*_ = < $I_{1}^{\prime }$, $I_{2}^{\prime }$, …, $I_{m}^{\prime }$>. Furthermore, assume without loss of generality that items in itemsets of sequences are sorted in lexicographical order. We can say that *S*_*a*_ matches *S*_*b*_ if (*S*_*a*_—*I*_1_) = (*S*_*b*_—$I_{m}^{\prime }$).

For example, <(*a*), (*c*, *e*), (*b*)> matches the two sequences <(*c*, *e*), (*b*, *d*)> and <(*c*, *e*), (*b*), (*d*)> but not the sequence of <(*e*, *c*), (*b*), (*d*)>.

**Definition 13 (S-Concatenation)** Let there be two *k*-sequences *S*_*a*_ and *S*_*b*_ such that *S*_*a*_ matches *S*_*b*_. If the last element $I_{m}^{\prime }$ in *S*_*b*_ has only one item in it, the sequence *S*_*ab*_ is generated by adding $I_{m}^{\prime }$ to *S*_*a*_, which is called the join of *S*_*a*_ with *S*_*b*_ by **S-Concatenation**.

For example, the sequence <(*a*), (*c*, *e*), (*b*)> matches the sequence <(*c*, *e*), (*b*), (*d*)>. The sequence <(*a*), (*c*, *e*), (*b*), (*d*)> is obtained by joining by **S-Concatenation** those two sequences.

**Definition 14 (I-Concatenation)** Let there be two *k*-sequences *S*_*a*_ and *S*_*b*_ such that *S*_*a*_ matches *S*_*b*_. If the last element $I_{m}^{\prime }$ in *S*_*b*_ has more than one item in it, the sequence *S*_*ab*_ is generated by adding the last item in $I_{m}^{\prime }$ to the last element in *S*_*a*_. This operation is called the join of *S*_*a*_ with *S*_*b*_ by **I-Concatenation**.

For example, the sequence <(*a*), (*c*, *e*), (*b*)> matches the sequence <(*c*, *e*), (*b*, *d*)>. The sequence <(*a*), (*c*, *e*), (*b*, *d*)> is obtained by joining the two previous sequences by **I-Concatenation**.

Given a set of *k*-sequences, (*k*+1)-sequences are generated as follows. If *k* is equal to 1, 2-sequences are generated. Each 1-sequence in the set is joined with itself using a **S-Concatenation** and is joined with all 1-sequences located after itself (according to the sorting order) both by **I-Concatenation** and **S-Concatenation**. Moreover, each sequence is joined with all 1-sequences located (sorted order) before itself by **S-Concatenation**. For example, consider the set of 1-sequences {<*a*>, <(*b*)>, <(*c*)>}. For the 1-sequence <(*b*)>, the following 2-sequences are generated <(*b*), (*b*)>, <(*b*, *c*)>, <(*b*), (*c*)>, and <(*b*), (*a*)>. For each *k*-sequence (*k* ≥ 2) *S*_*a*_ in the set, let *S*_*b*_ be a sequence succeeding *S*_*a*_ in the set, if *S*_*a*_ matches *S*_*b*_ and the last element in *S*_*b*_ has only one item, *S*_*a*_ is joined with *S*_*b*_ by **S-Concatenation**. If *S*_*a*_ matches *S*_*b*_ and the last element in *S*_*b*_ has more than one item, *S*_*a*_ is joined with *S*_*b*_ by **I-Concatenation**. For example, consider the set of 2-sequences {<(*ab*)>, <(*a*), (*b*)>, <(*b*, *c*)>, <(*b*), (*c*)>}. For the sequence <(*a*), (*b*)>, several sequences are generated including <(*a*), (*b*, *c*)>, <(*a*) and (*b*), (*c*)>.

Using the traditional level-wise approach for exploring the search space of patterns, it is necessary to evaluate patterns at each level, which is time consuming. We thus propose the following lemmas and pruning strategies to reduce the search space for mining the HUPSPs.

**Lemma 1** *The SWU of a sequence in an uncertain quantitative sequence database is greater than or equal to the SWU of any of its supersets*.

**Proof 1** *Let S^k^ be a k-sequence and S^k−1^ be one of its subsets*. *Since S^k−1^* ⊆ *S^k^*, *the set of sequence IDs (named SIDs) of S^k−1^ is a subset of the SIDs of S^k^*, *thus*:

$SWU(S^{k})=\Sigma_{S^{k}\subseteq S_{q}\wedge S_{q}\in USD}Su(S_{q})\leq \Sigma_{S^{k-1}\subseteq S_{q}\wedge S_{q}\in USD}=SWU(S^{k-1})$

$\Rightarrow SWU(S^{k})\leq SWU(S^{k-1}).$

Based on the above lemmas, we can obtain the following theorem.

**Theorem 1 (High probability downward closure property, HPDC property)** *The downward closure property holds for high probability sequential patterns*.

**Proof 2** *Let S^k^ be a k-sequence*, *and S^k−1^ be one of its sub-sequences*. *Thus*, *sp*(*S^k^*, *S_q_*) = *sp*(*S_q_*). *For any sequence S_q_ in USD*, *sp*(*S^k^*, *S_q_*) = *sp*(*S^k−1^*, *S_q_*). *Since S^k−1^ is a sub-sequence of S^k^*, *the set of SIDs of S^k−1^ is a subset of the SIDs of S^k^*. *Thus*,

$sp(S^{k})=\Sigma_{S^{k}\subseteq S_{q}\wedge S_{q}\in USD}\;sp(S_{q},\;S^{k})\leq \Sigma_{S^{k-1}\subseteq S_{q}\wedge S_{q}\in USD}=sp(S^{k-1})$

$\Rightarrow sp(S^{k})\leq sp(S^{k-1}).$

*Thus*, *if S^k^ is a HPSP*, *and its probability is no less than the minimum expected support sp*(*S^k^*) ≥ |*USD*| × *μ*, *then the subset S^k−1^ is a HPSP*.

**Corollary 1.** If a sequence *S*^*k*^ is a HPSP, each super-sequence *S*^*k*−1^ of *S*^*k*^ is also a HPSP.

**Corollary 2.** If a sequence *S*^*k*^ is not a HPSP, each non super-sequence *S*^*k*+1^ of *S*^*k*^ is a HPSP.

**Definition 15** A sequence *S* in a database *USD* is defined as a High Sequence-Weighted Utility-Probability Pattern (HSWUP) if 1) *sp*(*S*) ≥ |*USD*| × *μ*, and 2) *SWU*(*S*) ≥ *TSU* × *ε*.

In the designed algorithm, it recursively finds the set of HSWUPs with different lengths to maintain the downward closure property (or called HSWUPDC property) for later discovering the set of HUPSPs. For example, the set of 1-sequences is denoted as *HSWUPs*^1^ in the designed algorithm, and the set of 2-sequences is denoted as *HSWUPs*^2^ in the designed algorithm. Thus, the algorithm is to recursively find the set of *k*-sequences (*k* ≥ 1, denoted as *HSWUPs*^*k*^) until no candidates are generated (the set of (*k*-1)-sequences (*HSWUPs*^*k*−1^) becomes ***null***).

For example in Table 1, suppose that *μ* is set to 35%. The minimum expected support is calculated as 4 × 35% (= 1.4). Suppose that *ε* is set to 27.7%. The minimum utility count is calculated as 65 × 27.7% (= 18). A sequence <(*a*)> is a HSWPUP since its utility sequence-weighted utility (*SWU*) is *SWU*(<(*a*)>) = *SU*(*S*_1_) + *SU*(*S*_2_) + *SU*(*S*_4_) (= 17 + 16 + 17) (= 50 > 18), and its probability is *sp*(<(*a*)>) = *sp*(*S*_1_) + *sp*(*S*_2_) + *sp*(*S*_4_) (= 0.6 + 0.8 + 0.9) (= 2.3 > 1.4).

**Theorem 2 (High sequence-weighted utility-probability closure property, HSWUPDC property)** *Let S^k^ be a k-sequence*, *and S*^*k*−1^ *be one of its sub-sequence*, *where S*^*k*−1^ *is a HSWUP*. *The HSWUPDC property indicates that SWU*(*S*^*k*−1^) ≥ *SWU*(*S*^*k*^) *and sp*(*S*^*k*−1^) ≥ *sp*(*S*^*k*^).

**Proof 3** *Since S*^*k*−1^ ⊆ *S*^*k*^, *the set of SIDs of S*^*k*−1^ *is a subset of the SIDs of S*^*k*^. *Let S*^*k*^ *be a k-sequence*, *and S*^*k*−1^ *be one of its sub-sequences*. *It follows that*:

$SWU(S^{k})=\Sigma_{S^{k}\subseteq S_{q}\wedge S_{q}\in USD}\;SU(S_{q})\leq \Sigma_{S^{k-1}\subseteq S_{q}\wedge S_{q}\in USD}\;SU(S_{q})\Rightarrow SWU(S^{k-1})$

$\Rightarrow SWU(S^{k})\leq SWU(S^{k-1}).$

From **Theorem 1**, it can be found that *sp*(*S*^*k*^) ≤ *sp*(*S*^*k*−1^). Therefore, from Theorems 1 and 2, we have that if *S*^*k*^ is a HSWUP, *S*^*k*−1^ is also a HSWUP.

**Corollary 3.** If a sequence *S*^*k*^ is a HSWUP, every subset *S*^*k*−1^ of *S*^*k*^ is also a HSWUP.

**Corollary 4.** If a sequence *S*^*k*^ is not a HSWUP, each non super-sequence *S*^*k*+1^ of *S*^*k*^ is a HSWUP.

**Theorem 3 (HUPSPs ⊆ HSWUPs)** *The downward closure property of HSWUP ensures that HUPSPs* ⊆ *HSWUPs*. *It indicates that if a sequential pattern is not a HSWUP*, *none of its supersets are HUPSPs either HSWUPs*.

**Proof 4** ∀*S*^*k*^ ∈ *USD such that S*^*k*−1^ *is a (k-1)-sequence*. *We have that*:

*1*. *According to Theorem 2*, *we can obtain that if S*^*k*−1^ *is not a HSWUP*, *none of its supersets S*^*k*^ *will be a HSWUP*.

2. *Since* $su(S^{k-1})=\Sigma_{S^{k-1}\subseteq S_{q}\wedge S_{q}\in USD}\;su(S^{k-1},\;S_{q})\leq \Sigma_{S^{k-1}\subseteq S_{q}\wedge S_{q}\in USD}\;SU(S_{q})=SWU(S^{k-1}).$

$\Rightarrow su(S^{k-1})\leq SWU(S^{k-1}).$

*Thus*, *if S*^*k*−1^ *is not a HSWUP*, *S*^*k*−1^ *will not be a HUPSP nor a HSWUP*. *Any super-sequence S*^*k*^ *is neither a HSWUP nor a HUPSP*. *This theorem holds and is the basis that ensure the correctness and completeness of the proposed algorithm for mining HUPSPs*.

Based on the above definitions and theorems, three pruning strategies are developed as follows to reduce the search space for mining HUPSPs.

**Pruning strategy 1:** If the expected support and the *SWU* of a *k*-sequence *S*^*k*^ do not satisfy the two conditions: 1) *sp*(*S*^*k*^) ≥ |*USD*| × *μ*, and 2) *SWU*(*S*^*k*^) ≥ *TSU* × *ε*, this sequence can be directly pruned from the search space since none of its superset is a HUPSP.

**Rationale.** According to **Theorems 2 and 3**, this pruning strategy ensures that all HUPSP can be found.

**Pruning strategy 2:** Let *S*^*k*^ be a *k*-sequence. If any (*k*-1)-sub-sequence of the sequence *S*^*k*^ is not a HPSP, *S*^*k*^ and all its supersets are not HUPSPs.

**Rationale.** According to **Theorem 1**, this pruning strategy will not prune any HUPSPs.

**Lemma 2 (sub-sequence Utility Structure, SUS)** *If the SWU of a 2-sequence is less than the minimum utility count*, *its super-sequences are neither HSWUPs nor HUPSPs*.

**Proof 5** *Let S*^2^ *be a 2-sequence*, *and S*^*k*^ *be a k-sequence* (*k* ≥ 3) *that is a superset of S*^2^. *Thus*, *SWU*(*S*^*k*^) ≤ *SWU*(*S*^*k*−1^) *and HUPSPs* ⊆ *HSWPUPs hold*. *If SWU*(*S*^2^) < *TU* × *μ*, *S*^2^ *is not a HSWPUP and any superset of S*^2^ *w.r.t. a k-sequence is neither a HSWPUP nor a HUPSP*.

According to the definition of HUPSP, a HUPSP must satisfy two constraints by considering both the utility and probability measures (following the expected support model). Thus, if a sequence is not a HUSP, it is also not a HUPSP. A structure named sub-sequence Utility Structure (SUS) is built to store the *SWU* value of all 2-sequences. This structure can help us to avoid performing database scans and prune unpromising candidates early. From the given example in Table 1, the built SUS is shown in Table 4.

**Table 4:** The built SUS for the running example.

Sequence	SWU	Sequence	SWU
<(*a*), (*a*)>	50	<(*b*, *c*)>	31
<(*a*, *b*)>	33	<(*b*), (*c*)>	34
<(*a*), (*b*)>	50	<(*c*), (*c*)>	0
<(*a*, *c*)>	17	<(*c*), (*b*)>	33
<(*a*), (*c*)>	17	<(*c*), (*a*)>	33
<(*b*), (*b*)>	33	<(*b*), (*a*)>	50

Based on the SUS, the following pruning strategy is obtained.

**Pruning strategy 3:** Let *S*^*k*^ be a *k*-sequence (*k* ≥ 3). If the *SWU* of a 2-sequence *S* ⊆ *S*^*k*^ and its SWU is less than than the minimum utility count found in SUS, *S*^*k*^ is not a HSWUP and is not a HUPSP. Moreover, none of its super-sequences are HUPSPs.

**Rationale.** According to **Lemmas 1 and 3**, this pruning strategy is correct. The reason is that since HUPSPs ⊆ HSWUPs, thus if a 2-sequence *S* ⊆ *S*^*k*^ holds *SWU*(*S*) ≤ *STU* × *ε*, *S* is not a HSWUP. Moroever, *S* and all its super-sequences are not HUPSPs. Hence, using the SUS pruning strategy, a huge number of unpromising sequences can be pruned early.

Based on the above definitions and theorems, the following process for mining HUPSPs is proposed, which is divided into two phases. In the first phase, the designed baseline U-HUSPM algorithm performs a breadth-first search to mine the complete set of HSWUPs. Based on the HSWUPDC property, if the probability value (expected support) of a sequential pattern is less than the minimum expected support count or its SWU value is less than the minimum utility count, this pattern and all its supersets are not HSWUPs, and can thus be pruned in the search space. In the second phase, an additional database scan is performed to identify the actual HUPSPs from the set of HSWUPs discovered in the first phase. The pseudo-code of the proposed algorithm is shown in Algorithm 1.

**Algorithm 1**: Baseline U-HUSPM

 **Input:** *USD*, an uncertain quantitative sequence database; *ptable*, a unit profit table; *ε*, minimum utility threshold; *μ*, minimum expected support threshold.

 **Output:** HUPSPs, a set of complete high utility-probability sequential patterns

1 **for** each *i*_*j*_ ∈ *USD* **do**

2  scan *USD* to calculate *SWU*(*i*_*j*_) and *sp*(*i*_*j*_)

3 calculate *TSU* of *USD*;

4 **for** each *i*_*j*_ ∈ *USD* **do**

5  **if** *sp*(*i*_*j*_) ≥ |*USD*| × *μ* ∧ *SWU*(*i*_*j*_) ≥ *TSU* × *μ* **then**

6   *HSWUPs*^1^ ← *HSWUPs*^1^∪*i*_*j*_;

7 set *k* ← 2;

8 **while** *HSWUPs*^*k*−1^≠ ***null* do**

9  *C*_*k*_ = *genCand*(*HSWUPs*^*k*−1^);

10  **for** each *S*^*k*^ ∈ *C*_*k*_ **do**

11   **if** ∃*s* ⊆ *S*^*k*^ ∧ *sp*(*s*)< |*USD*| × *μ* ∥ *SWU*(*s*) < *TSU* × *ε* **then**

12    continue;;

13  calculate *SWU*(*S*^*k*^) and *sp*(*S*^*k*^);

14  **if** *sp*(*S*^*k*^) ≥ |*USD*| × *μ* ∧ *SWU*(*S*^*k*^) ≥ *STU* × *ε* **then**

15   *HSWUPs*^*k*^ ← *HSWUPs*^*k*^ ∪ *S*^*k*^;

16  *HSWUPs* ← *HSWUPs* ∪ *HSWUPs*^*k*^;

17  *k* ← *k* + 1;

18 **for** each *S* ∈ *HSWUPs* **do**

19  **if** *su*(*S*) ≥ *TSU* × *ε* **then**

20   *HUPSPs* ← *HUPSPs*∪*S*;

21 return *HUPSPs*;

The proposed U-HUSPM algorithm takes as input: (1) an uncertain sequential database USD, (2) a unit profit table ptable indicating the unit profit of each item, (3) the minimum utility threshold *ε*, and (4) the minimum expected support threshold *μ*. The algorithm performs the following steps. First, the database is scanned to calculate the SWU and the expected support of each item (Lines 1 to 2). In the running example, 1-sequences are *sp*(*a*) = 2.3, *sp*(*b*) = 2.8, *sp*(*c*) = 0.8, *sp*(*d*) = 1.3, *sp*(*e*) = 0.6, *sp*(*f*) = 0.5 and *SWU*(*a*) = 50, *SWU*(*b*) = 65, *SWU*(*c*) = 65, *SWU*(*d*) = 31, *SWU*(*e*) = 17, *SWU*(*f*) = 15.

For each 1-sequence, the designed algorithm first checks whether the SWU and the expected support of each 1-sequence satisfies the conditions and keep the HSWUPs (Lines 4 to 6). In the running example, 1-sequences <(*a*)>, <(*b*)> and <(*c*)> are considered as the HSWUPs and put into the set of *HSWUP*^1^. The parameter *k* is then set to 2 (Line 7), and a loop is performed to discover all HSWUPs using a level-wise approach (Lines 8 to 17). Notice that *k* is defined as the length of the sequences. For example, *k* is defined as 1 (*k* = 1) for representing the (*k* = 1)-sequences; *k* is defined as 2 (*k* = 2) for representing (*k* = 2)-sequences. The designed U-HUSPM algorithm first generates the set of 2-sequence candidates by joining 1-sequences in the set of *HSWUPs*^1^.

During the (*k*-1)-th iteration of the loop, the set of *HSWUP*^*k*^ is obtained by the following process. First, pairs of (*k*-1)-sequences in *HSWUP*^*k*^ are joined to generate their super-sequences of length *k* by applying the generate-and-test procedure using **I-Concatenations** and **S-Concatenations**. Details of this process was given in Definitions 14 and 15. The pruning strategies 2 and 3 are applied to prune unpromising sequences early, and thus reduce the search space. For example, the built SUS shown in Table 4 can be used to easily obtain the *SWU* values of each 2-sequence. Using this structure, the algorithm can easily prune unpromising candidates without rescanning the database. If the SWU of a sequence is no less than the minimum utility count and its expected support count is no less than the minimum expected support count, this sequence is put into the set *HSWUP*^*k*^. For example, consider the sequence *<*(*a*), (*b*), (*c*)*>*. It is not necessary to scan the database to calculate the *SWU* values of the 2-sequences of <(*a*), (*c*)> since it does not satisfy the condition (the *SWU* value is less than the minimum utility count).

The loop is repeated until no HSWUPs are generated. After that, an additional database scan is performed to find the actual HUPSPs from the sequences in the set of HSWUPs (Lines 18 to 20). Finally, the set of HUPSPs is returned as the final result (Line 21). For the running example, the final result is shown in Table 3.

### 0.1 An improved projection model

Since the baseline algorithm applies a level-wise approach to mine HUPSPs, it can have long execution times and consumes a huge amount of memory. To overcome this problem, this section proposes an improved algorithm named P-HUSPM. The P-HUSPM algorithm utilizes the projection mechanism to project a database into smaller databases for each processed sequence. Using this approach, the runtime and memory usage can be reduced.

**Definition 16** Let there be two sequences *S*_*a*_ = <*I*_1_, *I*_2_, …, *I*_*n*_> and *S*_*b*_ = <$I_{1}^{\prime }$, $I_{2}^{\prime }$, …, $I_{m}^{\prime }$> (1 ≤ *m* ≤ *n*). Moreover, assume that all items in each element in sequences are lexicographically ordered. The sequence *S*_*b*_ is called a prefix of *S*_*a*_ iff 1) $I_{i}^{\prime }=I_{i}$ (1 ≤ *i* ≤ *m*-1); 2) $I_{m}^{\prime }\subseteq I_{m}$ and all items in ($I_{m}-I_{m}^{\prime }$) are lexicographically ordered after those in *I*_*m*_.

For example, <*a*>, <(*a*), (*c*)> and <(*a*), (*c*, *e*), (*b*)> are prefixes of the sequence <(*a*), (*c*, *e*), (*b*, *d*)> but <(*a*), (*e*)> and <(*a*), (*c*, *e*), (*d*)> is not.

**Definition 17** Let there be two sequence *S* and *S*_*q*_ such that *S* ⊆ *S*_*q*_. A sub-sequence of the sequence *S*_*q*_ is called a projected sequence of *S* if (1) the sequence has prefix *S* and (2) no proper super-sequence of *S* such that the sequence is a sub-sequence of *S*_*q*_ having *S* as prefix. This relationship is denoted as *S*_*q*_|*S*. Thus, the projected database of a sequence *S* in an uncertain database *USD* is the collection of all projected sequences of each sequence in the database corresponding to the sequence *S*, denoted by *USD*|*S*.

For example, the projected sequence of <(*a*), (*b*), (*a*, *c*, *e*)> of (*a*) is <(*b*), (*a*, *c*, *e*)>. For the running example of Table 1, the projected database of the sequence <(*a*)> is shown in Table 5.

**Table 5:** The projected database of <(*a*)>.

SID	Sequence	Expected Support	SU
1	<(*a*, 3), (*b*, 4), [(*a*, 1), (*c*, 1)]>	0.6	15
2	<[(*a*, 1)], [(*a*, 2), (*b*, 2)]>	0.8	8
3	<[(*a*, 1), (*b*, 2)], [(*a*, 4), (*b*, 1)]>	0.9	13

The pseudo-code of the P-HUSPM algorithm is shown in Algorithm 2.

**Algorithm 2:** Projection HUSPM, P-HUSPM

 **Input:** *USD*, an uncertain quantitative sequence database; *ptable*, a unit profit table; *ε*, minimum utility threshold; *μ*, minimum expected support threshold.

 **Output:** HUPSPs, a set of complete high utility-probability sequential patterns

1 **for** each *S*_*q*_ ∈ *USD* **do**

2  scan *USD* to calculate *SU*(*S*_*q*_);

3 **for** each *i*_*j*_ ∈ *USD* **do**

4  calculate *sp*(*i*_*j*_) and *SWU*(*i*_*j*_);

5 *HSWUPs*^1^ ←{<(*i*_*j*_) > |*SWU*(*i*_*j*_) ≥ *TSU* × *ε* ∧ *sp*(*i*_*j*_) ≥ |*USD*| × *μ*};

6 remove *i*_*j*_ ∉ *HSWUPs*^1^ from *USD* as *USD*’;

7 **for** each 1-sequence *S* ∈ *HSWUPs*^1^ **do**

8  calculate *su*(*S*);

9  **if** *su*(*S*) ≥ *TSU* × *ε* **then**

10   *HUPSPs* ← *S*;

11  **Mining** (*USD*’, *ptable*, *ε*, *μ*, *HSWUPs*^1^)

The P-HUSPM algorithm takes as input (1) *USD*, an uncertain quantitative sequence database, (2) *ptable*, a unit profit table, (3) *ε*, the minimum utility threshold, and (4) *μ*, the minimum expected support threshold. First, each sequence is scanned to find its sequence utility (Lines 1 to 2). For each individual item *i*_*j*_ (1-sequence) in *USD*, the sequential-weighted utility (*SWU*) and expected support count are respectively calculated (Lines 3 to 4). If the *SWU* of the sequence is no less than the minimum utility count and its expected support is no less than the minimum expected support count, the sequence is then put into the set of *HSWPUP*^1^ (Line 5). In this example, 1-sequences satisfying this condition are <(*a*)>, <(*b*)> and <(*c*)>, which will be put into the set of *HSWUPs*^1^. A database scan is performed to remove items that do not appear in the set of *HSWPUP*^1^ and the revised database *USD*’ is then obtained (Line 6). For example, the sequence <(*a*)> is a HSWUP and its projected database is shown in Table 5.

For each 1-sequence in *HSWPUP*^1^, the sequential utility is calculated. If the utility is no less than the minimum utility threshold, this sequence is output as a HUPSP (Lines 9 to 10). Finally, the sub-process **Mining**(*USD*’, *ptable*, *ε*, *μ*, *HSWPUP*^1^) is recursively executed to discover all HUPSPs (Line 11). The pseudo-code of the **Mining** process is shown in Algorithm 3. For the running example, the final result is shown in Table 3.

**Algorithm 3:** Mining(*USD*’, *ptable*, *ε*, *μ*, *HSWUPs*^*k*^)

 **Input:** *USD*, an uncertain quantitative sequence database; *ptable*, a unit profit table; *ε*, minimum utility threshold; *μ*, minimum expected support threshold; *HSWUPs*^*k*^, the discovered HSWUPs of *k* length.

 **Outut:** HUPSPs, a set of complete high utility-probability sequential patterns

1 **for** each sequence *S*^*k*^ ∈ *HSWUPs*^*k*^ **do**

2  project sub-database *USD*′|*S*^*k*^;

3  *HSWUPs*^*k*+1^ ← ***null***;

4  **for** each *S*^*k*+1^ of *S*^*k*^ in *USD*′|*S*^*k*^ **do**

5   calculate *SWU*(*S*^*k*+1^), *sp*(*S*^*k*+1^);

6   **if** *SWU*(*S*^*k*+1^) ≥ *TSU* × *ε*∧*sp*(*S*^*k*+1^) ≥ |*USD*| × *μ* **then**

7    *HSWUPs*^*k*+1^ ← *HSWUPs*^*k*+1^ ∪ *S*^*k*+1^;

8   calculate *su*(*S*^*k*+1^) from *HSWUPs*^*k*+1^;

9   **for** each *s* ∈ *S*^*k*+1^ **do**

10    **if** *su*(*s*) ≥ *TSU* × *ε* **then**

11     *HUPSPs* ← *HUPSPs* ∪ *s*;

12  **Mining** (*USD*′|*S*^*k*^, *ptable*, *ε*, *μ*, *HSWUPs*^*k*+1^);

The **Mining** process takes as input: (1) *USD*’, a refined uncertain quantitative sequence database, (2) *ptable*, a unit profit table indicating the unit profit of each item, (3) *ε*, the minimum utility threshold, (4) *μ*, the minimum expected support threshold, and (5) *HSWUPs*^*k*^, the HSWUPs of length *k*. For each *k*-sequence *S*^*k*^ in *HSWUPs*^*k*^, a database scan is performed to generate the projected database *USD*′|*S*^*k*^ (Line 2). For the running example, the extended sequences of sequence <(*a*)> are <(*a*), (*a*)>, <(*a*, *b*)>, <(*a*), (*b*)>, <(*a*, *c*)> and <(*a*), (*c*)>. An empty set *HSWUPs*^*k*+1^ is then created (Line 3). The *USD*′|*S*^*k*^ is scanned once to find the *SWU* and *sp* of *S*^*k*+1^ by performing **I-Concatenations** and **S-Concatenations** (Lines 4 to 5). If the *SWU* of *S*^*k*+1^ is no less than the minimum utility count and its expected support count value is no less than the minimum expected support count, this sequence will be added to the set *HSWUPs*^*k*+1^ (Lines 6 to 7). The actual utility of this sequence is then calculated and the HUPSPs are output (Lines 8 to 11). This loop is repeated until all sequences in *HSWUPs*^*k*^ have been processed (Line 12).

## 1 Experimental evaluation

Substantial experiments were conducted to evaluate the effectiveness and efficiency of the proposed algorithms. Two versions of the baseline algorithm named U-HUSPM1 and U-HUSPM2 are considered. They respectively apply pruning strategies 1 and 2 (U-HUSPM1), and pruning strategies 1 to 3 (U-HUSPM2). The P-HUSPM algorithm adopts not only pruning strategies 1 to 3 but also the projection mechanism to reduce the size of projected databases, which can greatly reduce the time required for processing a dataset. Notice that this is the first paper where mining high utility-probability sequential patterns in uncertain databases is considered. Hence, no previous work can be directly compared to the proposed algorithms. Experiments were performed on a personal computer having an Intel(R) Core(TM) i7-4790 CPU @3.60GHz and 8GB of RAM, running the 64-bit Microsoft Windows 7 operating system. All algorithms were implemented using the Java language.

Experiments were carried on several real-life datasets, having various characteristics. All tested databases were obtained from the SPMF website [36]. The BMS and the kosarak10k datasets are both sparse datasets containing a few very long sequences.

SIGN is a dense dataset containing numerous very long sequences. LEVIATHAN and the FIFA are moderately dense datasets, having many long sequences. BIBLE is a moderately dense dataset having many medium length sequences. For all tested datasets, the log-normal distribution was used to generate the external utilities of all items in sequences in the range of 0 and 1000. The purchase quantities of items was randomly generated in the range of 1 and 5. The probability of all sequences were randomly generated in the 0 and 1 interval. In summary, the parameters describing these datasets are: #|**D**|, the number of sequences, #|**I**|, the number of distinct items, **AvgLen**, the average sequence length, **MaxLen**, the maximal sequence length, and **Type**, the database type. Characteristics of the datasets used in the experiments are presented in Table 6. The datasets used in the experiments are provided in http://ikelab.net/db/plosone_db.rar.

**Table 6:** Characteristics of the datasets.

Dataset	#|D|	#|I|	AvgLen	MaxLen	Type
SIGN	730	267	52	94	sign language
LEVIATHAN	5,834	9,025	33.8	100	book
FIFA	20,450	2,990	36.2	100	click-stream
BIBLE	36,369	13,905	21.6	100	book
kosarak10k	10,000	10,094	8.1	608	click-stream
BMS	59,601	497	2.5	267	click-stream

To assess the performance of the developed algorithms, the runtime, number of generated candidates, memory usage and scalability were evaluated. In each experiment, an algorithm was terminated if its runtime exceeded 10,000 seconds or if it ran out of memory.

### 1.2 Runtime

**Fig 1:**
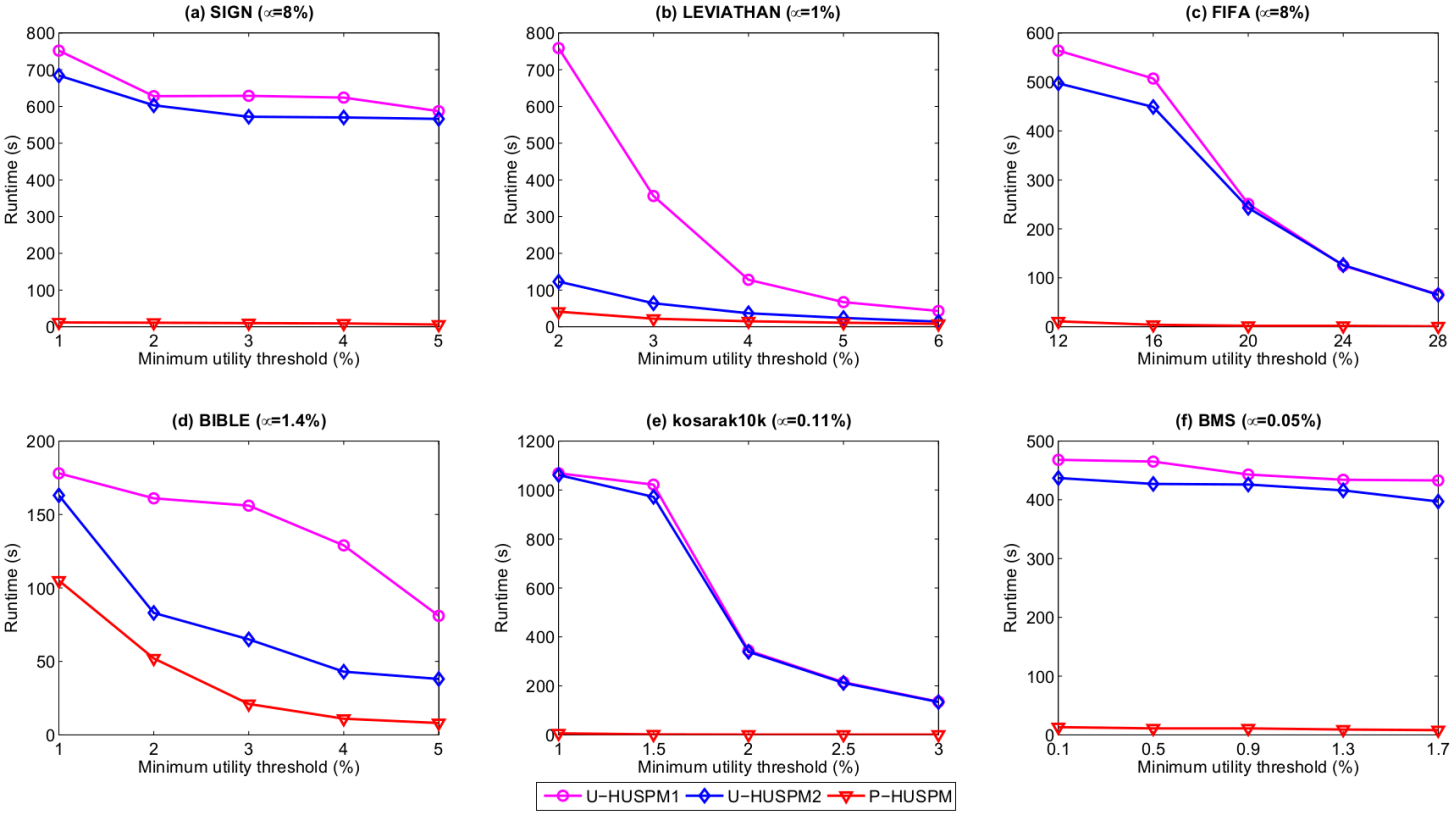
Runtimes when *μ* is fixed and *ε* is varied.

In this section, the runtimes of the proposed U-HUSPM1, U-HUSPM2, and P-HUSPM algorithms are compared. Fig 1 shows the runtime of the proposed algorithms when the minimum expected support threshold *μ* is fixed and the minimum utility threshold *ε* is varied within a predefined interval for each database.

**Fig 2:**
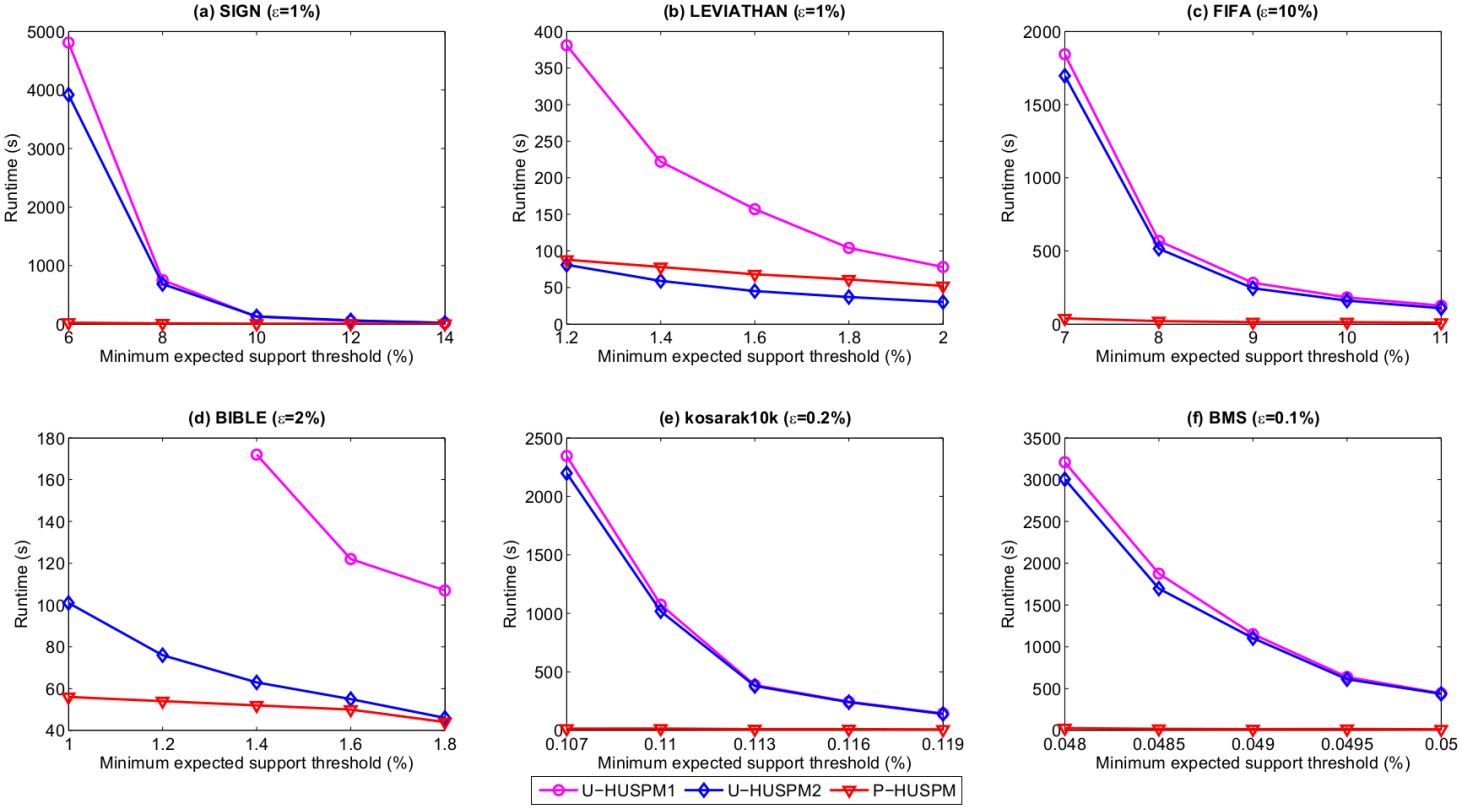
Runtimes for a fixed *ε* and various *μ* values.

It can be observed in Fig 1 that the proposed P-HUSPM algorithm outperforms U-HUSPM1 and U-HUSPM2 on the six datasets for a fixed minimum expected support threshold when the minimum utility threshold is varied. When *μ* is set to 8% and *ε* is set to 12% on the FIFA dataset, for example, the runtimes of U-HUSPM1, U-HUSPM2 and P-HUSPM are respectively 564, 497 and 11 seconds. A reason is that P-HUSPM uses the projection mechanism, which can reduce the size of databases for processed sequences. For large sequences, projected databases are smaller. P-HUSPM thus outperforms the U-HUSPM1 and U-HUSPM2 algorithms. It can also be observed that the U-HUSPM2 algorithm has better performance than that of the U-HUSPM1 algorithm for most datasets. The reason is that the U-HUSPM2 algorithm adops pruning strategies 1 to 3, while the U-HUSPM1 algorithm only applies the pruning strategies 1 and 2. Pruning strategy 3 can prune huge amounts of unpromising sequences early to avoid performing multiple database scans for calculating the *SWU* and expected support of sequences. For example, when *μ* is set to 1.4% and *ε* is set to 2% on the BIBLE dataset, the runtimes of U-HUSPM1 and U-HUSPM2 are respectively 161 and 83 seconds. It can also be observed that the performance gap between U-HUSPM1 and U-HUSPM2 is large for the SIGN, LEVIATHAN, FIFA and BIBLE datasets especially when *μ* is set to small values. The reason is that these four datasets are dense, and many correlated HUPSPs are found. As a result, pruning strategy 3 can be used to prune unpromising HSWUPs early. In addition, Fig 2 shows the runtime of the proposed algorithms for a fixed *ε* and various *μ* values.

It can be observed that P-HUSPM and U-HUSPM2 are faster than U-HUSPM1 on the six datasets for mining the HUPSPs. The reasons are that P-HUSPM uses the projection mechanism to reduce the size of the projected databases, and the U-HUSPM2 adopts all the designed pruning strategies to reduce the size of the search space. Moreover, as the minimum expected support threshold *μ* is increased, the three algorithms spend less time to discover the HUPSPs. The reason is that as *μ* is increased, less candidates are generated for mining the HUPSPs. It can be also observed in Fig 2 that the performance gap between U-HUSPM1 and U-HUSPM2 is greater for the SIGN, LEVIATHAN, FIFA and BIBLE datasets. The reason is the same as in Fig 1. Thus, the proposed pruning strategy 3 can greatly improve the performance of the baseline U-HUSPM algorithms to discover the HUPSPs.

### 1.2 Number of candidates

**Fig 3:**
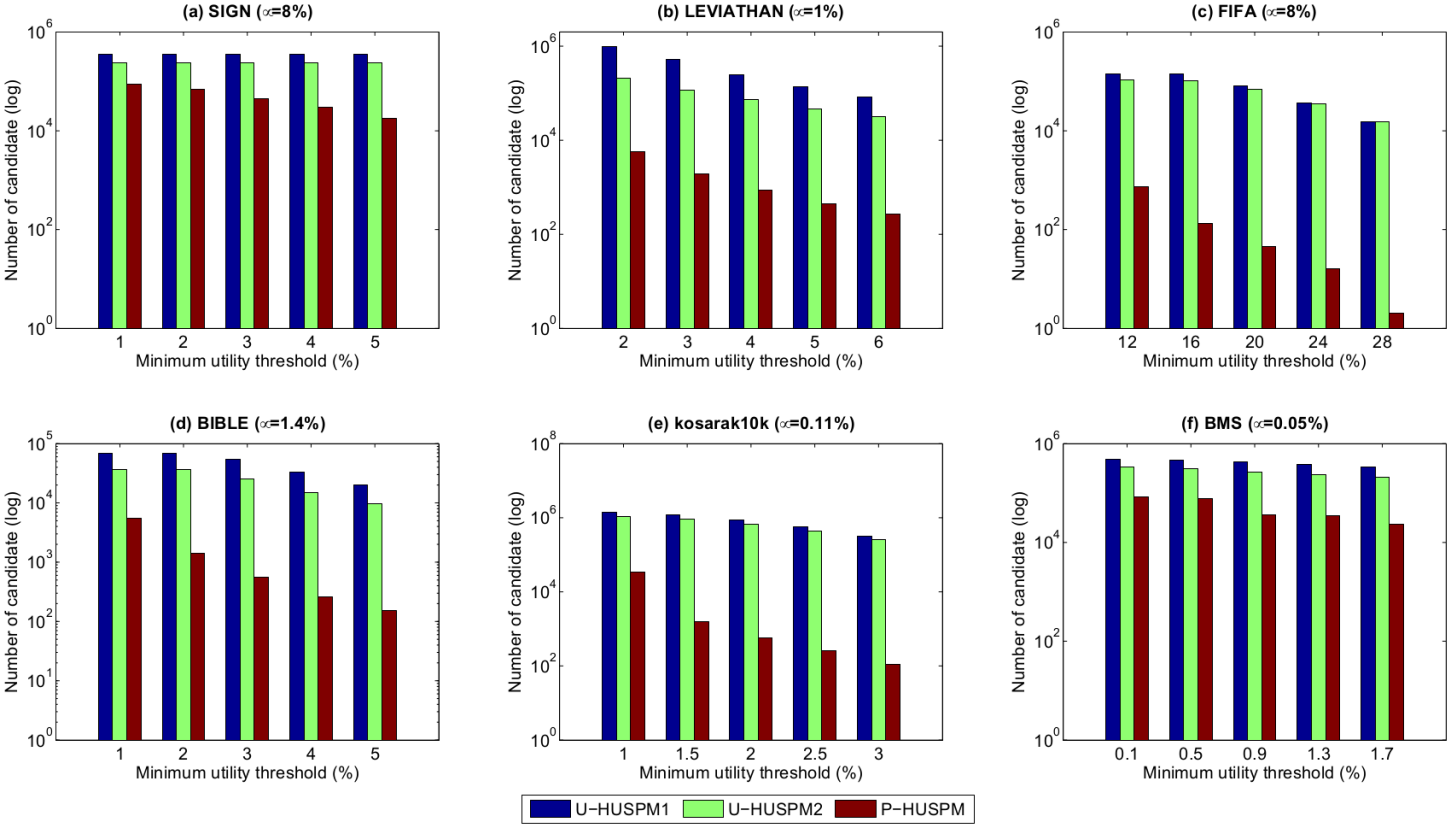
Number of candidates for a fixed *μ* when *ε* is varied.

**Fig 4:**
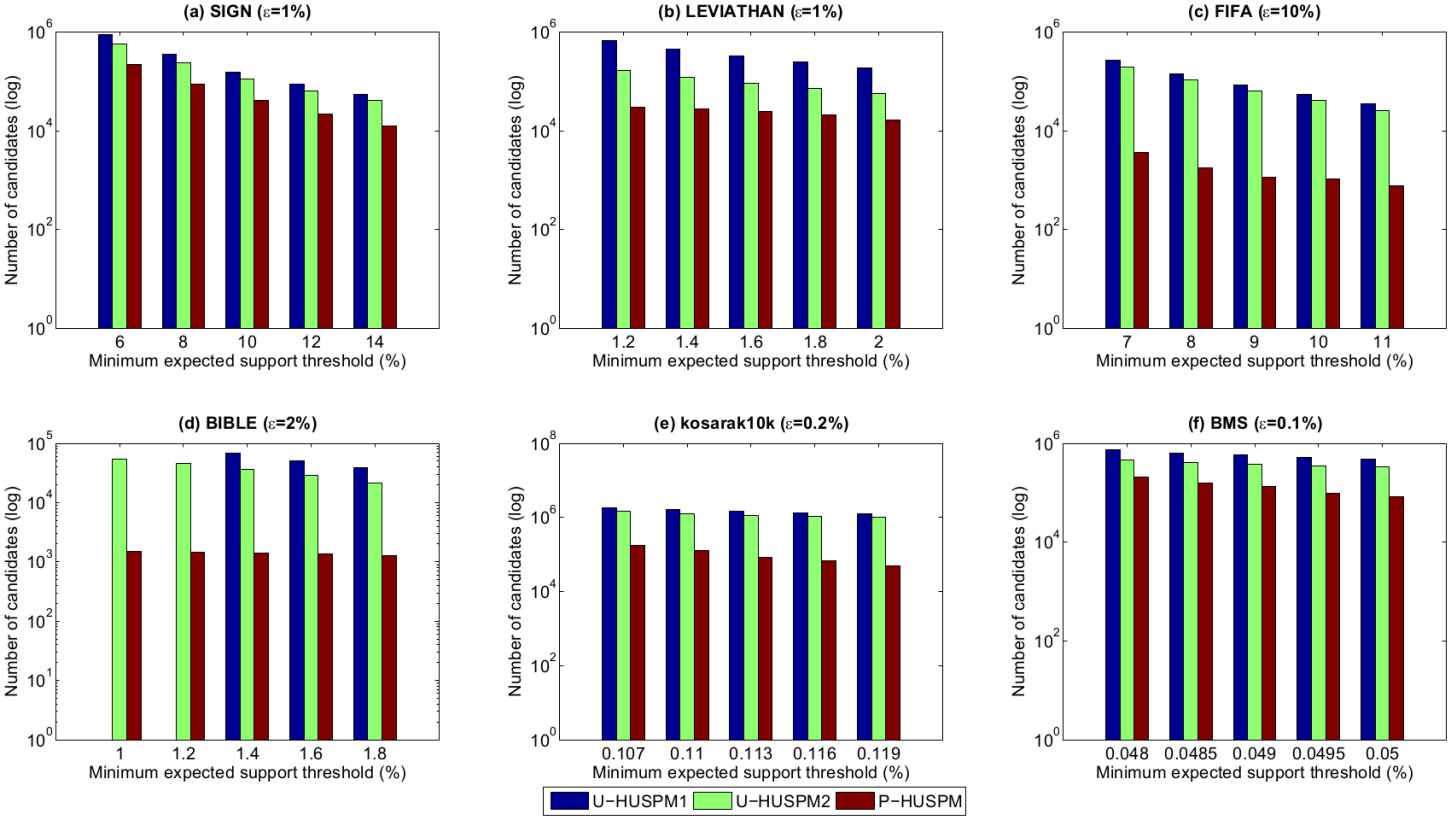
Number of candidates for a fixed *ε* when *μ* is varied.

This section evaluates the performance in terms of number of candidates generated by each of the three algorithms. A sequence is considered to be a candidate if it requires an additional database scan to determine its actual utility. Fig 3 shows the number of candidates for the three algorithms for a fixed *μ* when the minimum utility threshold *ε* is varied. Fig 4 shows the number of candidates generated by the three algorithms for a fixed *ε* when the minimum utility threshold *μ* is varied.

In Figs 3 and 4, it can be found that the number of candidates decreases as the minimum utility threshold is increased or as the minimum expected support threshold is increased. The reason is that when the minimum expected support threshold or minimum utility threshold are increased, fewer candidates are generated for mining the HUPSPs. It is also obvious to see that the P-HUSPM algorithm always generates less candidates than the other two algorithms. For example, when *μ* is set to 8% and *ε* is set to 1% on the SIGN database, the number of candidates for U-HUSPM1, U-HUSPM2 and P-HUSPM are respectively 347,396, 234,841 and 87,309. The reason is that the P-HUSPM algorithm adopts the projection mechanism to generate a small database for each processed sequence. Thus, the *SWU* value of a processed sequence is much smaller than that obtained by the baseline U-HUSPM1 and U-HUSPM2 algorithms. The projection mechanism can be used to greatly reduce the size of the processed candidates for mining the final set of HUPSPs. It also can be observed in Fig 4 that the U-HUSPM2 algorithm always generates less candidates than the U-HUSPM1 algorithm. The reason is that pruning strategy 3 is used in U-HUSPM2 to reduce the number of candidates.

### 1.3 Memory usage

**Fig 5:**
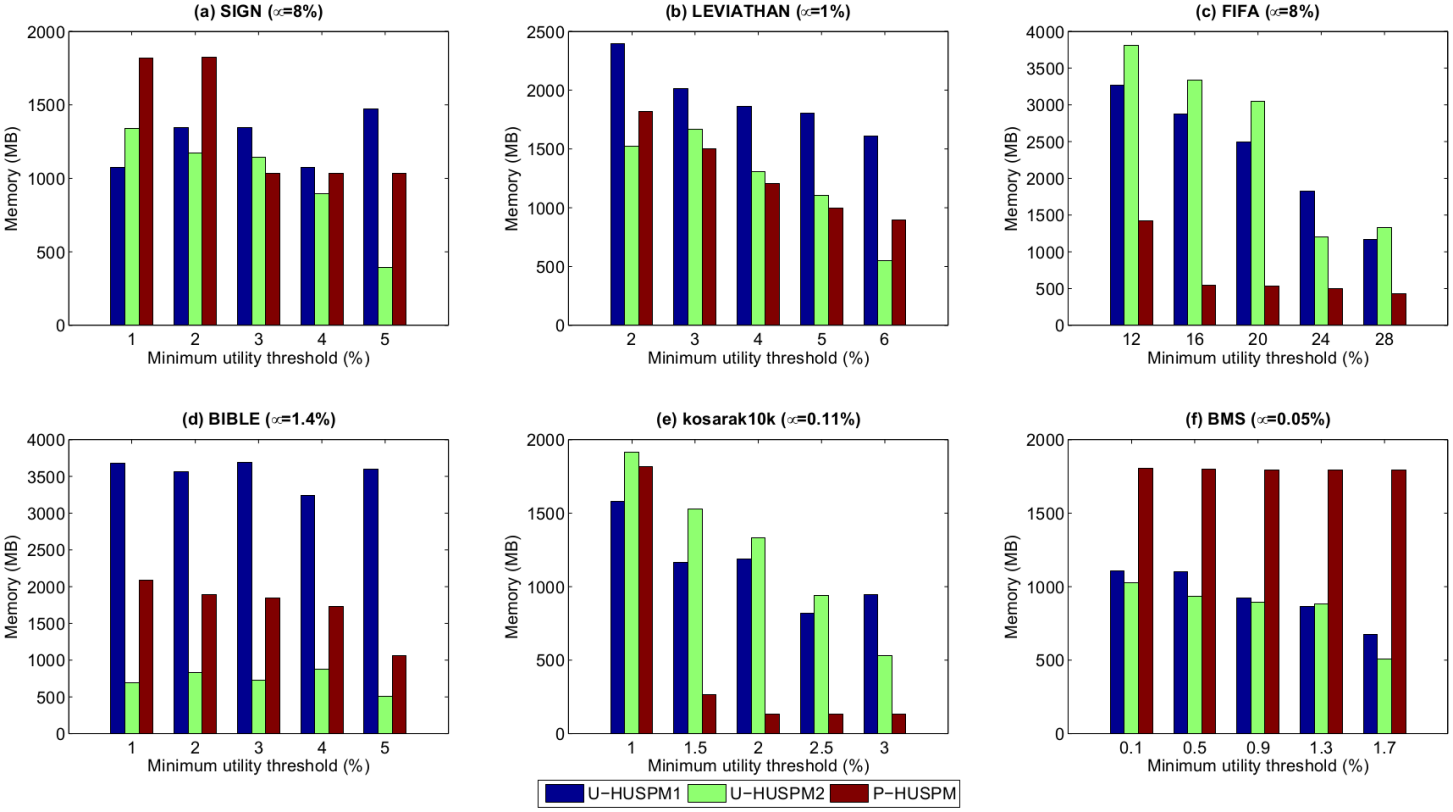
Memory usage for a fixed *μ* when *ε* is varied.

**Fig 6:**
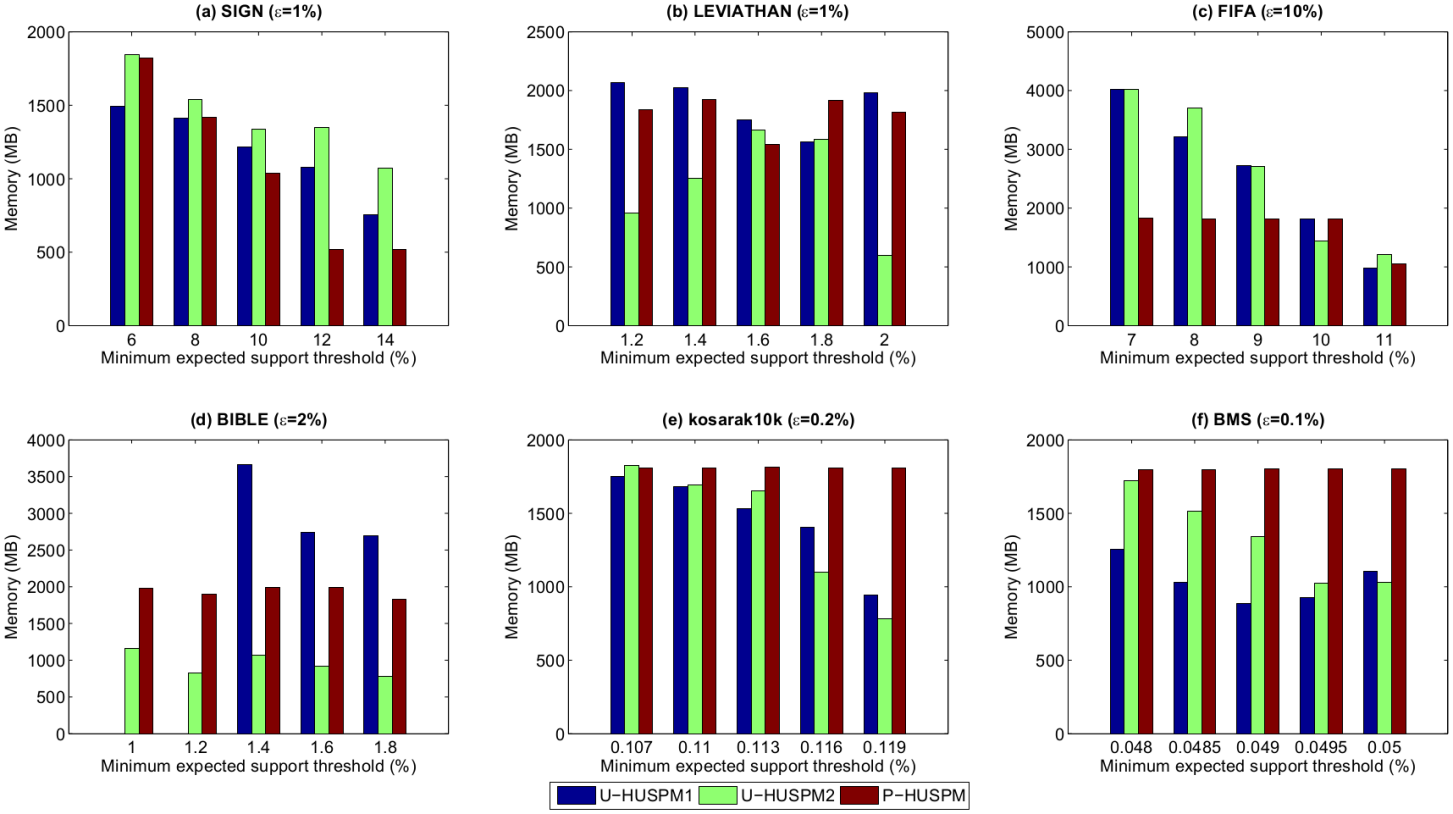
Memory usage for a fixed *ε* when *μ* is varied.

Experiments were also carried out to assess the memory usage of the compared algorithms. Fig 5 shows the results for a fixed *μ* when *ε* is varied, while Fig 6 shows the results for a fixed *ε* when *μ* is varied.

In most cases, the U-HUSPM2 algorithm consumes less memory than the U-HUSPM1 and P-HUSPM algorithms. The P-HUSPM algorithm applies the projection mechanism to project a database for each processed sequence. Thus, this process requires more memory. However, P-HUSPM performs better than U-HUSPM2 in Fig 5(c) and 5(e). The reason is that those two datasets are sparse. When the projection mechanism is performed, a much smaller database is obtained for a given sequence. Since the U-HUSPM2 algorithm adopts the pruning strategy 3 to reduce the number of candidates, its memory usage is greatly reduced even though an extra (but few) arrays are used to store the relationships of 2-sequences. For the U-HUSPM2 algorithm, it is unnecessary to project and store the projected databases of a processed sequence. Thus less memory is used compared to the P-HUSPM algorithm in most cases. Thus, the U-HUSPM2 algorithm always requires less memory compared to the U-HUSPM1 and P-HUSPM algorithms. The U-HUSPM1 algorithm does not adopt pruning strategy 3 to efficiently reduce the search space for mining the required information. Hence, more memory is required for handling unpromising candidates. Although the P-HUSPM algorithm adopts pruning strategies 1 to 3, it sometimes needs extra memory to perform projections, especially for dense datasets or when a sequence has a high chance of having almost the same utility and probability in a sparse dataset. There is thus a trade-off between memory usage and runtime. Although, P-HUSPM sometimes requires more memory than U-HUSPM1 (in Figs 5(f), 6(e) and 6(f)), P-HUSPM is faster than U-HUSPM1 algorithm. For the U-HUSPM2 algorithm, it can be seen that it generally outperforms U-HUSPM1, as well as P-HUSPM in terms of memory usage except for very sparse datasets such as FIFA and kosarak.

### 1.4 Scalability

**Fig 7:**
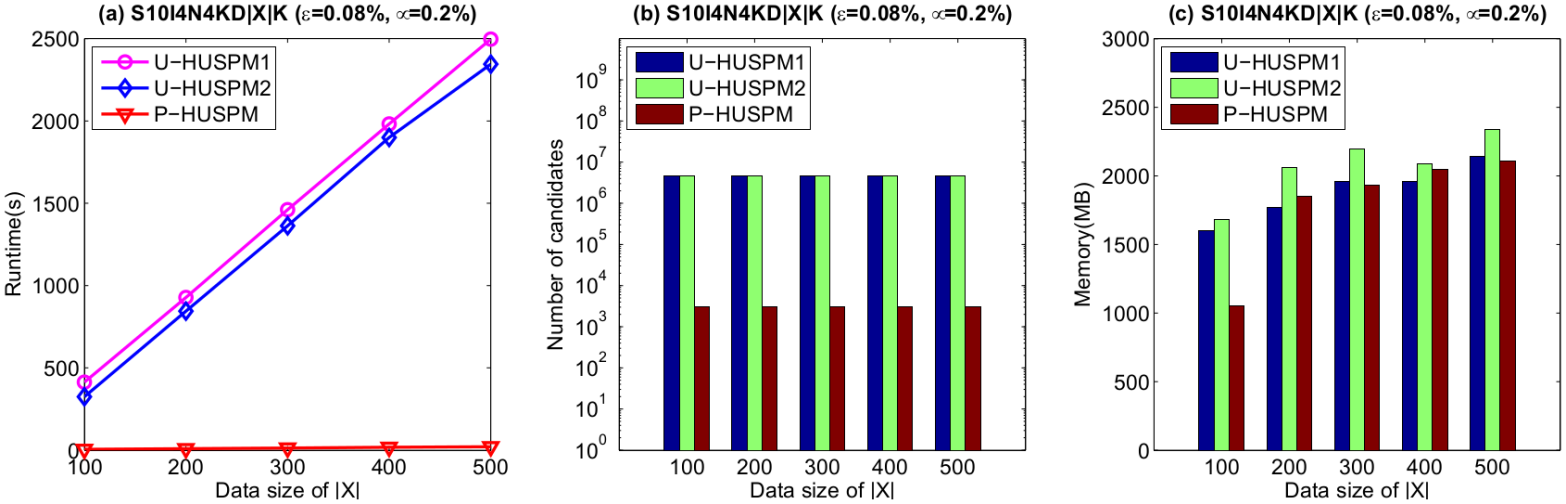
Scalability of the compared algorithms.

The scalability of the proposed algorithms was also evaluated. Experiments were performed on a series of synthetic datasets named S10I4N4KD|X|K, where the number of sequences *X* was varied from 100k to 500k sequences using increments of 100k. Since there is no dataset available for the designed framework yet, the parameters that we used for generating sequential dataset were obtained from the literature [7, 35–38]. Those parameters are commonly used for generating synthetic datasets. The minimum utility threshold is set to 0.08%, while the minimum probability threshold is set to 0.2%. Results in terms of runtime, number of candidates and memory usage are shown in Fig 7.

In Fig 7, it can be seen that the P-HUSPM algorithm has better scalability compared to the other two algorithms, especially in terms of runtime. It also can be observed that the runtime and memory usage increases as database size is increased, but that the number of candidates remains steady. It can be also easily observed that the number of candidates for the three algorithms remain stable for different dataset sizes. However, the runtime and the memory usage increase since the developed algorithms needs more runtime to calculate the *SWU* and the expected support of sequences. This process also consumes more memory to keep the required information for mining the HUPSPs.

## 2 Conclusion

In the past, several studies have proposed algorithms to mine high utility sequential patterns in precise data but no studies were published to handle uncertain databases for mining the high utility-probability sequential patterns. In this paper, two baseline algorithms named U-HUSPM and P-HUSPM were respectively presented to efficiency and effectively mine the high utility-probability sequential patterns (HUPSPs) in uncertain databases. The U-HUSPM algorithm mines the HUPSPs using a level-wise approach, and applies three pruning strategies to reduce the number of unpromising candidates in the search space. Besides, an improved P-HUSPM algorithm was designed. Its projection mechanism allows to create small projected databases for each processed sequence, which speeds up the mining process. Substantial experiments were conducted on both synthetic and real datasets to evaluate the performance of the developed algorithms in terms of runtime, number of candidates, memory usage and scalability. Results have shown that the proposed algorithms and the designed pruning strategies can efficiently discover HUPSPs and the correctness and completeness has been demonstrated.

Since this is the first paper to level-wisely mine the HUPSPs, more extensions with better structures (such in [22, 23]) can be also considered to efficiency improve the mining performance. However, it is a non-trivial task since the downward closure property used in the varied data structures is necessary to be maintained and re-designed, as well as the pruning strategies to early reduce the unpromising candidates and reduce the search space for mining HUPSPs. Moreover, the designed algorithm in this paper mines HUPSPs. However, the utility of a sequence increases along with the size of it. The average-utility model can be used to provide a fair measurement regarding to the length of the itemset/sequence (number of items within it). It is also an interesting topic to mine the high average-utility sequences under uncertain databases.
